# Interstitial cortisol levels obtained by adipose tissue microdialysis in mechanically ventilated septic patients: correlations with total and free serum cortisol

**DOI:** 10.1186/cc9831

**Published:** 2011-03-11

**Authors:** M Theodorakopoulou, N Nikitas, I Ilias, M Tzanela, D Vassiliadi, P Kopterides, N Maniatis, A Diamantakis, S Orfanos, I Perogamvros, A Armaganidis, U Ungerstedt, K Keevil, T Tsagarakis, D Dimopoulou

**Affiliations:** 1 Attikon University Hospital, Athens, Greece; 2 Alexandra Hospital, Athens, Greece; 3 E. Venizelou Hospital, Athens, Greece; 4 Evagelismos Hospital, Athens, Greece; 5 Christie Hospital, Manchester, UK; 6 Karolinska Institute, Stockholm, Sweden; 7 University Hospital of South Manchester, Manchester, UK; 8 Polyclinic Hosital, Athens, Greece

## Introduction

The aim of this study was to measure cortisol in the interstitial fluid of mechanically ventilated septic patients using MD and to examine the correlation between interstitial cortisol levels and total along with free serum cortisol.

## Methods

A prospective study including 31(20 men) septic patients. All patients met the ACCP/SCCM criteria for sepsis. Upon sepsis an MD catheter was inserted in the subcutaneous tissue of the upper thigh. MD sampling was done on days 1 and 2, six times/day. The collected samples were analyzed for free cortisol, glucose, pyruvate, lactate, glycerol and lactate/pyruvate ratio. Blood samples were collected for routine hematology and biochemistry on the same days. Age, gender, sepsis stage, administration of vasopressors, death in the ICU and 28-day mortality were recorded. APACHE II scores for day 1 and SOFA scores for days 1 and 2 were calculated.

## Results

Seventeen patients were given norepinephrine. Albumin on day 1 was uniformly low. One-third of patients died. Cortisol values in the interstitial fluid remained constant (*P *= 0.480). Serum total cortisol (*P *= 0.116) and serum total cortisol/albumin ratio (*P *= 0.127) were also constant. On day 2 serum-free cortisol was higher than MD-free cortisol. Log MD cortisol correlated strongly with the log serum total cortisol and serum-free cortisol on day 2 correlated well with serum total cortisol. Day 1 log MD cortisol correlated positively with log MD pyruvate and log APACHE II. Day 2 log MD cortisol correlated positively with norepinephrine dose and log SOFA score. There were no other significant correlations of MD cortisol.

## Conclusions

Adipose tissue cortisol is strongly correlated with serum total and free cortisol, suggesting that serum cortisol reflects tissue cortisol availability. The utility of MD in studying cortisol dynamics needs to be further investigated.

